# Conventional Versus Traction-Assisted Endoscopic Submucosal Dissection for Esophageal, Gastric, and Colorectal Neoplasms: A Systematic Review and Meta-Analysis of Randomized Controlled Trials

**DOI:** 10.7759/cureus.55645

**Published:** 2024-03-06

**Authors:** Felipe Giacobo Nunes, Igor Logetto Caetité Gomes, Diogo Turiani Hourneaux De Moura, Juan Eduardo G Dominguez, Fernando Fornari, Igor Braga Ribeiro, Guilherme Henrique Peixoto de Oliveira, Sérgio Mazzola P de Figueiredo, Wanderley Marques Bernardo, Eduardo G Hourneaux de Moura

**Affiliations:** 1 Gastrointestinal Endoscopy, Hospital das Clínicas da Faculdade de Medicina da Universidade de São Paulo, São Paulo, BRA; 2 Gastroenterology, Federal University of Fronteira Sul, Passo Fundo, BRA; 3 Surgery, Cleveland Clinic Center for Abdominal Core Health, Cleveland Clinic Foundation, Cleveland, USA

**Keywords:** endoscopic advanced treatment, gastrointestinal endoscopy, endoscopy, traction, endoscopic submucosal dissection

## Abstract

Endoscopic submucosal dissection (ESD) is increasingly being utilized for the resection of superficial gastrointestinal neoplasms. However, the long procedure time poses a technical challenge for conventional ESD (C-ESD). Traction-assisted ESD (T-ESD) was developed to facilitate the procedure by reducing its duration. This study compares the efficacy and safety of C-ESD versus T-ESD in the treatment of esophageal, gastric, and colorectal neoplasms. Nine randomized controlled trials (RCTs) were analyzed. Traction-assisted ESD exhibited shorter mean dissection times for the esophagus and colorectal regions and lower perforation rates in colorectal cases. No significant differences were observed in en bloc resection or bleeding rates. Traction-assisted ESD proves to be more efficient in mean procedure time for esophageal and colorectal cases and safer in perforation rates for colorectal cases, but similar rates are noted for en bloc resection or bleeding.

## Introduction and background

Superficial gastrointestinal neoplasms, which are defined in most guidelines as lesions limited to the mucosa or submucosa without invading the muscularis propria, regardless of the presence of lymph node involvement, are susceptible to endoscopic treatment using advanced techniques such as endoscopic submucosal dissection (ESD). Lesions located in the esophagus, stomach, or colorectal region can be effectively and safely resected en bloc. In addition, this technique provides a less invasive approach than surgical procedures. However, ESD has the limitation of being technically difficult, requiring a long procedure time, and having a long learning curve [1].

Conventional ESD (C-ESD) is a technique performed in three phases: delimitation, incision, and dissection. A wide variety of devices have been developed to assist in its performance, such as dual electrosurgical knives. These accessories assist in the dissection stage by allowing both the injection of solutions to elevate the submucosa and the cutting of tissue [2]. However, the tubular shape of the endoscopic device and the difficulty in manipulating the tissues in a non-axial way limit the operator's movements and can prolong the procedure time [3].

Traction-assisted ESD (T-ESD) emerged as a method to facilitate the dissection of the space between the mucosa and muscularis propria by increasing the separation of the planes exerted by traction [4,5]. Different techniques have been developed to exert this traction, either with external traction or internal devices. The most commonly used are clips with thread, S-O clips (traction clips), ring-shaped thread, clip-flaps, grasping forceps, dental floss, springs, and loops [6, 7].

This systematic review and meta-analysis aimed to compare the efficacy and safety of C-ESD versus T-ESD in the approach to neoplasms of the esophagus, stomach, and colorectal region. Efficacy was analyzed by mean dissection time and en bloc resection rate, and safety by perforation and bleeding rate. 

## Review

Methods 

Study Protocol and Registration

This meta-analysis was reported according to the guidelines of Preferred Reporting Items for Systematic Reviews and Meta-Analyses (PRISMA) [8]. The study protocol was registered at the International Prospective Register of Systematic Reviews (PROSPERO) (registration number: CRD42022385314).

Search Strategy

An electronic search of the Medical Literature Analysis and Retrieval System Online (MEDLINE), Excerpta Medica database (Embase), and Cochrane Central Register of Controlled Trials (CENTRAL) databases was performed, as was a manual search of the references of the most relevant studies. The combination of the following operators was used to perform the search: “endoscopic submucosal dissection," "ESD," "complete endoscopic resection," “submucosal tunneling endoscopic resection," “endoscopic full-thickness resection," "traction," “thread-traction," "clip,” and "randomized controlled trial." Detailed search strategies can be found below in Table 1.

**Table 1:** Detailed electronic database search strategy MEDLINE: Medical Literature Analysis and Retrieval System Online; Embase: Excerpta Medica database; CENTRAL: Cochrane Controlled Register of Trials

Databases	Electronic search strategy
MEDLINE	((ESD) OR (Endoscopic Submucosal Dissection) OR (Dissection, Endoscopic Submucosal) OR (Submucosal Dissection, Endoscopic) OR (Endoscopic Full Thickness Resection) OR (Endoscopic Mucosal Resection) OR (Submucosal Tunneling Endoscopic Resection)) AND ((Traction) or (Rubber Band) or (Thread-traction) or (clip)) AND (Randomized Controlled Trials)
Embase	(‘ESD' OR 'Endoscopic Submucosal Dissection' OR 'Dissection, Endoscopic Submucosal' OR 'Submucosal Dissection, Endoscopic' OR 'Endoscopic Full Thickness Resection' OR 'Endoscopic Mucosal Resection' OR 'Submucosal Tunneling Endoscopic Resection’) AND (‘Traction' or 'Rubber Band' or 'Thread-traction' or ‘clip’) AND ('randomized controlled trial'/exp OR 'randomized controlled trial’)
CENTRAL	((ESD) OR (Endoscopic Submucosal Dissection) OR (Dissection, Endoscopic Submucosal) OR (Submucosal Dissection, Endoscopic) OR (Endoscopic Full Thickness Resection) OR 'Endoscopic Mucosal Resection' OR 'Submucosal Tunneling Endoscopic Resection’) AND (‘Traction' or 'Rubber Band' or 'Thread-traction' or ‘clip’) AND ('randomized controlled trial'/exp OR 'randomized controlled trial’)

The searches were carried out between January and May 2023. There were no restrictions on language or period of publication. Only full texts were included. Alerts were created in the databases informing of new results.

Eligibility Criteria

The eligibility criteria for this study included adults diagnosed with neoplasms of the esophagus, stomach, or colorectal region undergoing either C-ESD or T-ESD. The outcomes of interest comprised mean dissection time, en bloc resection rates, perforation, and bleeding. Only randomized controlled trials (RCTs) were considered eligible for inclusion. Exclusion criteria encompassed studies with overlapping patients, experimental animal studies, and conference abstracts.

Study Selection and Data Collection

Two independent reviewers carried out all the steps. Initially, they were responsible for excluding duplicate studies identified in the different databases. Subsequently, they read the titles and abstracts of the remaining studies, selecting those that addressed the subject of the study. Finally, the full texts were read, and those that met the inclusion criteria were selected. A consensus was established between the reviewers in the presence of disagreements at any stage of the selection. Data were also extracted independently by two reviewers and recorded on specific collection forms. Disagreements at the data collection stage were resolved by consensus between the reviewers after retrieving the information in the original article.

Risk of Bias and Quality of Studies

The risk of bias was assessed individually for each study using the Cochrane Risk of Bias 2 (RoB 2) tool [9]. The quality of the evidence was assessed using the Grading of Assessment, Development, and Evaluation of Recommendations (GRADE) [10].

Outcomes

The primary outcome analyzed was efficacy, which is defined by the en bloc resection rate and the mean dissection time. The secondary outcome was safety, which was analyzed by adverse effects, including the rate of perforation and bleeding.

Statistical Analysis

The data on dissection time were presented as a mean with a standard deviation in relation to the total number of procedures carried out. The rates of en bloc resection, perforation, and bleeding were presented as a proportion of the absolute number of events in relation to the total number of procedures performed. Data were presented using pooled and subgroup analyses for the esophagus, stomach, and colorectal location for each of the outcomes.

Heterogeneity was assessed by the Statistical Inconsistency Index (I2). Values <30%, 30%-60%, 61%-75%, and >75% were considered low, moderate, high, and very high, respectively. The fixed effect model was used for low/moderate values. If I2 was considered high/very high, we carried out a sensitivity analysis using a funnel plot to identify possible outliers. If the sample became homogeneous after excluding possible outliers, the studies were permanently excluded (considered true outliers), and a fixed effects model was used. When there were no outliers or heterogeneity remained high after excluding outliers, we used random effects to reduce the influence of heterogeneity on the final result.

The results were expressed as risk differences (RDs) with the respective confidence intervals (95% CI). Intention-to-treat analysis was performed in all studies. Statistical analyses were carried out using Review Manager (RevMan), version 5.4.1 (The Cochrane Collaboration, 2020).

Results

Study Selection

**Figure 1:**
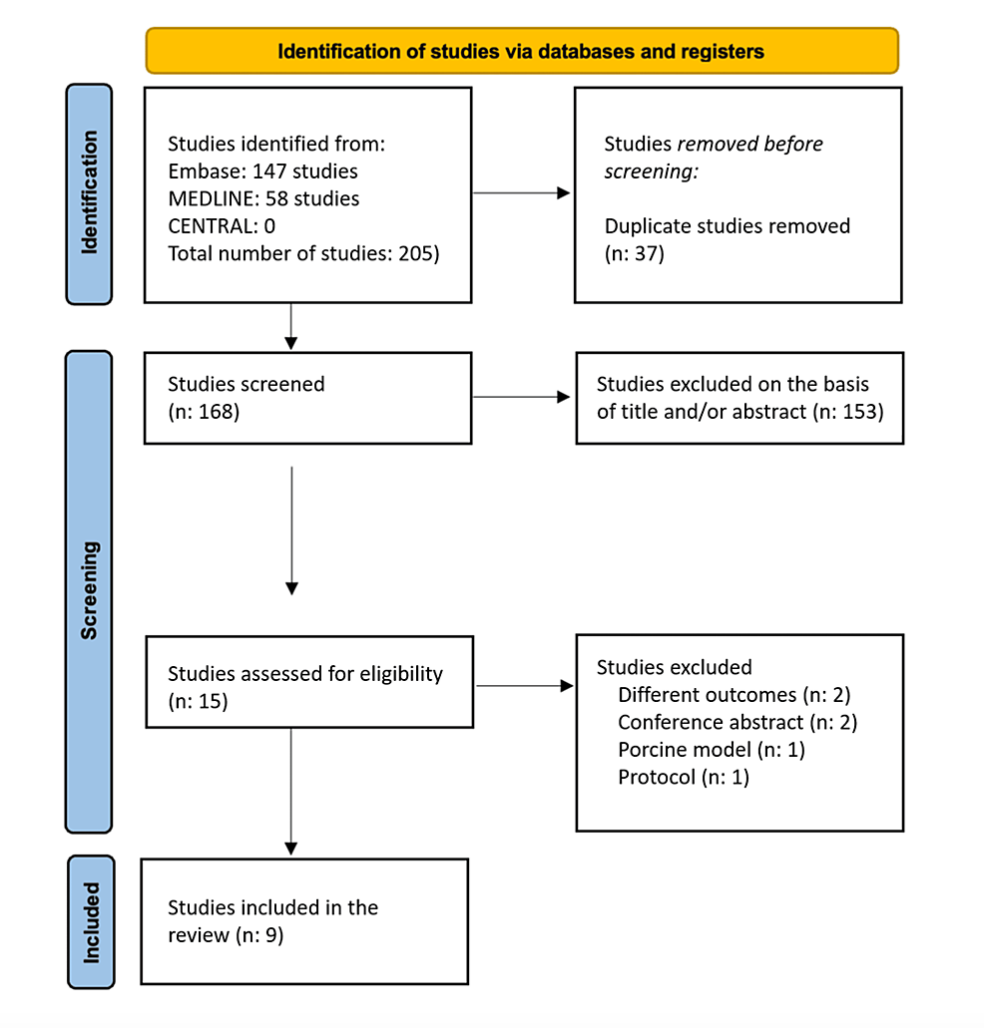
A flowchart outlining the screening process for study inclusion in the meta-analysis based on PRISMA guidelines Out of 205 initially identified studies, 37 duplicates were removed. Following the title and abstract review, 153 studies were excluded. A full-text assessment of the remaining 15 articles was conducted based on pre-established inclusion and exclusion criteria, resulting in the selection of nine studies for meta-analysis inclusion. PRISMA: Preferred Reporting Items for Systematic Reviews and Meta-Analyses; Embase: Excerpta Medica database;  MEDLINE: Medical Literature Analysis and Retrieval System Online; CENTRAL: Cochrane Central Register of Controlled Trials

A total of 205 studies were identified. Of these, 37 were duplicates, and 153 were excluded after reading the titles and abstracts. The remaining 15 articles were read in full text and subjected to inclusion and exclusion criteria, which enabled nine studies to be selected to compose this meta-analysis. A detailed flowchart of the study selection process is shown in Figure 1.

Study Characteristics

A total of 1,477 patients were analyzed. Of these, 741 patients underwent C-ESD and 736 underwent T-ESD. One study was Chinese [11], and all others were Japanese [12-19]. Among them, two evaluated procedures were performed in the esophagus [12, 13], three in the stomach [14-16], and four in the colorectal location [11,17-19]. Regarding traction techniques, five studies used clip methods [12-14, 17, 18], one combined clip with dental floss [15], one used the ring-thread technique [19], one used grasping forceps [11], and the other used the spring and loop for traction [16]. The detailed characteristics of the included studies are presented in Table 2. 

**Table 2:** Characteristics of the included studies T-ESD: traction-assisted endoscopic submucosal dissection; C-ESD: conventional endoscopic submucosal dissection; RCT: randomized controlled trial

Author and year	Patients (n) T-ESD vs. C-ESD	Location of the lesion	Country	Traction method	Type of study
Koike et al., 2015 [12]	20/20	Esophagus	Japan	Clip with thread	RCT
Ritsuno et al., 2014 [17]	27/23	Colon	Japan	S-O clip	RCT
Mori et al., 2017 [19]	21/22	Colon	Japan	Ring-shaped thread	RCT
Ban et al., 2018 [14]	49/55	Gastric	Japan	Clip–flap	RCT
Wang et al., 2019 [11]	21/20	Colon	China	Grasping forceps	RCT
Yoshida et al., 2018 [15]	319/316	Gastric	Japan	Dental floss	RCT
Yoshida et al., 2020 [13]	116/117	Esophagus	Japan	Clip–flap	RCT
Nagata et al., 2021 [16]	40/40	Gastric	Japan	Spring and loop	RCT
Ichijima et al., 2022 [18]	123/128	Colon	Japan	Clip–flap	RCT

Risk of Bias and Quality of Studies

The application of the RoB2 tool identified that two selected studies [12,17] presented a low risk of bias due to the appropriate randomization process, the presence of all outcome data with their correct measurement, and the absence of deviations in the intended interventions or the selection of the reported outcome. One study [16] presented some concerns in the outcome measurement domain and six studies [11,13-15,18,19] in the domain of the selection of the reported outcome. A detailed description of the risk of bias is presented below in Table 3.

**Table 3:** Risk of bias in the included studies

Study ID	Randomization process	Deviations from intended interventions	Missing outcome data	Measurement of the outcome	Selection of the reported results
Koike et al., 2015 [12]	Low risk	Low risk	Low risk	Low risk	Low risk
Ritsuno et al., 2014 [17]	Low risk	Low risk	Low risk	Low risk	Low risk
Mori et al., 2017 [19]	Low risk	Low risk	Low risk	Low risk	Some concerns
Ban et al., 2018 [14]	Low risk	Low risk	Low risk	Low risk	Some concerns
Wang et al., 2019 [11]	Low risk	Low risk	Low risk	Low risk	Some concerns
Yoshida et al., 2018 [15]	Low risk	Low risk	Low risk	Low risk	Some concerns
Yoshida et al., 2020 [13]	Low risk	Low risk	Low risk	Low risk	Some concerns
Nagata et al., 2021 [16]	Low risk	Low risk	Low risk	Some concerns	Low risk
Ichijima et al., 2022	Low risk	Low risk	Low risk	Low risk	Some concerns

The quality of the evidence in the included studies revealed a high degree of certainty for the evidence, which is presented in Appendix 1.

Mean Dissection Time

**Figure 2:**
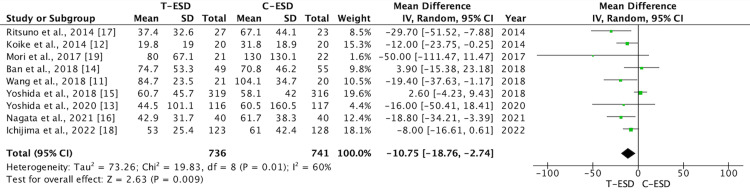
A forest plot of dissection time T-ESD: traction-assisted endoscopic submucosal dissection with traction); C-ESD: conventional endoscopic submucosal dissection

A total of 1,477 patients were analyzed for this outcome, with 741 undergoing C-ESD and 736 undergoing T-ESD in the nine studies included. The T-ESD group had a shorter mean dissection time than the C-ESD group (mean difference: -10.75; 95% CI (-18.76, -2.74); p = 0.009; I2 = 60%) (Figure 2).

**Figure 3:**
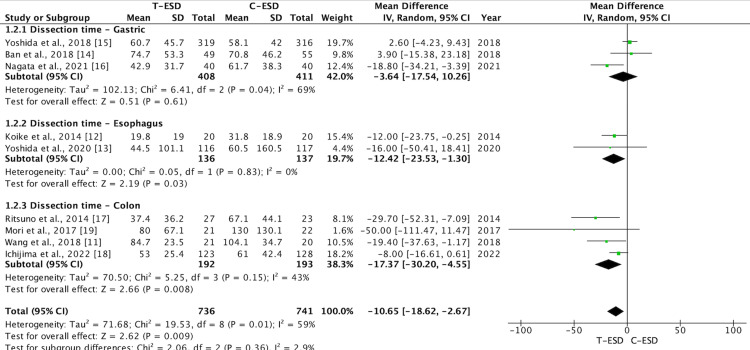
A forest plot of procedure time: subgroup analysis T-ESD: traction-assisted endoscopic submucosal dissection with traction); C-ESD: conventional endoscopic submucosal dissection

When subgroup analysis was carried out, this difference was maintained for the esophagus (mean difference: -12.42; 95% CI (-23.53, -1.30); p = 0.03; I2 = 0%) and colorectal regions (mean difference: -17.37; 95% CI (-30.20, -4.55); p = 0.008; I2 = 43%). There was no difference in the analysis of the stomach subgroup (mean difference: -3.64; 95% CI (-17.54, 10.26); p = 0.61; I2 = 69%) (Figure 3).

The GRADE analysis revealed a high certainty of evidence.

En Bloc Resection Rate

**Figure 4:**
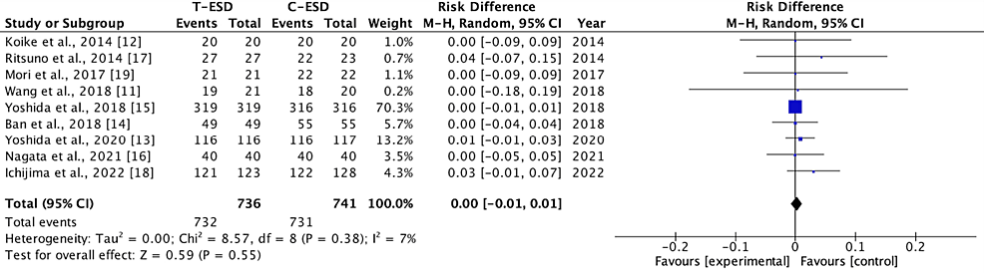
A forest plot of en bloc resection rate T-ESD: traction-assisted endoscopic submucosal dissection with traction); C-ESD: conventional endoscopic submucosal dissection

All nine studies evaluated the outcome of the en bloc resection rate. There was no difference in this outcome when comparing the C-ESD with the T-ESD in the pooled analysis (RD: <0.00; 95% CI (-0.01, 0.01); p = 0.55; I2 = 7%) (Figure 4).

**Figure 5:**
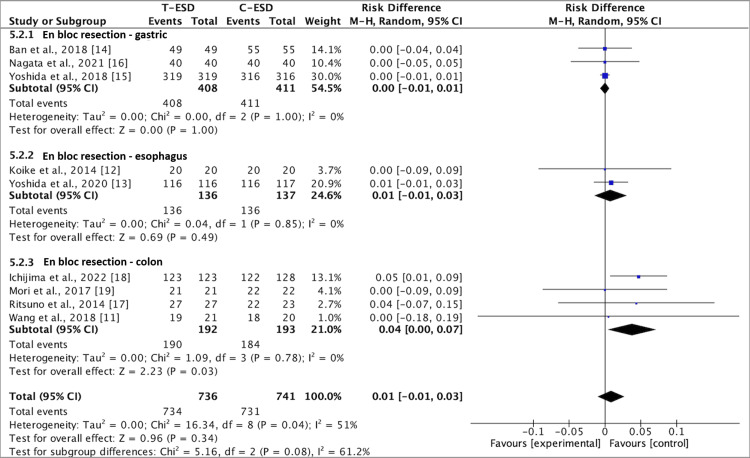
A forest plot of en bloc resection rate: subgroup analysis T-ESD: traction-assisted endoscopic submucosal dissection with traction); C-ESD: conventional endoscopic submucosal dissection

In the subgroup analysis of the esophagus (RD: 0.01; 95% CI (-0.01, 0.03); p = 0.85; I2 = 0%) and stomach (RD, 0.00; 95% CI (-0.01, 0.01); p = 1.00; I2 = 0%), there was also no difference between C-ESD and T-ESD, but in the analysis of the colorectal region subgroup (RD: 0.04; 95% CI (0.00, 0.07); p = 0.78; I2 = 0%), a difference favorable to the T-ESD method was identified (Figure 5).

The GRADE analysis revealed a high certainty of evidence.

Perforation Rate

**Figure 6:**
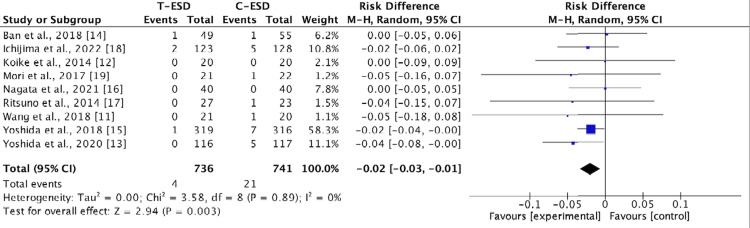
A forest plot of perforation rate T-ESD: traction-assisted endoscopic submucosal dissection with traction); C-ESD: conventional endoscopic submucosal dissection

This outcome was assessed by the nine studies, and the pooled analysis showed that T-ESD had a lower perforation rate than C-ESD (RD: -0.02; 95% CI (-0.03, -0.01); p = 0.89; I2 = 0%) (Figure 6).

**Figure 7:**
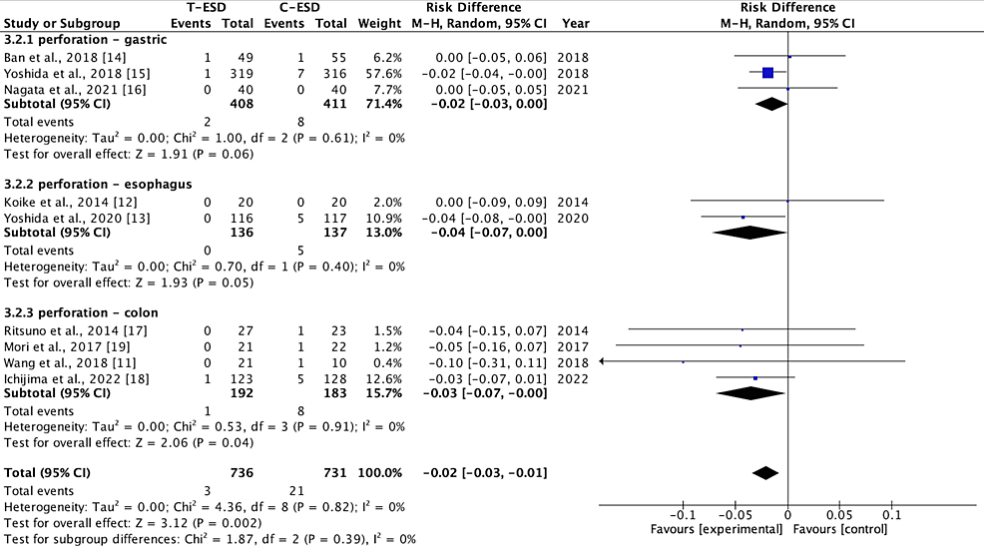
A forest plot perforation rate: subgroup analysis T-ESD: traction-assisted endoscopic submucosal dissection with traction); C-ESD: conventional endoscopic submucosal dissection

This difference was maintained in the subgroup analysis for the colorectal region (RD: -0.03; 95% CI (-0.07, <-0.00); p = 0.04; I2 = 0%). There was no difference in the analysis of the esophagus subgroup (RD: -0.04; 95% CI (-0.07, >0.00); p = 0.05; I2 = 0%) and the stomach subgroup (RD: -0.02; 95% CI (-0.03, >0.00); p = 0.06; I2 = 0%) (Figure 7).

The GRADE analysis revealed a high certainty of evidence.

Bleeding Rate

**Figure 8:**
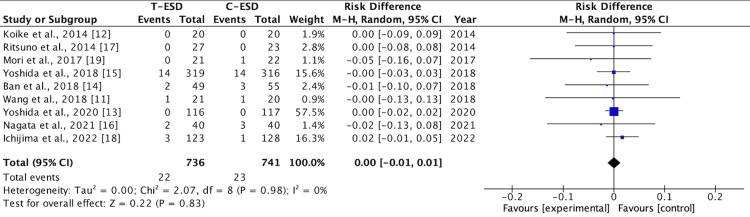
A forest plot of the bleeding rate T-ESD: traction-assisted endoscopic submucosal dissection with traction); C-ESD: conventional endoscopic submucosal dissection

Bleeding rates were reported in the nine included studies. There was no difference in this outcome when comparing C-ESD with T-ESD in the pooled analysis (RD: <0.00; 95% CI (-0.01, 0.01); p = 0.83; I2 = 0%) (Figure 8).

**Figure 9:**
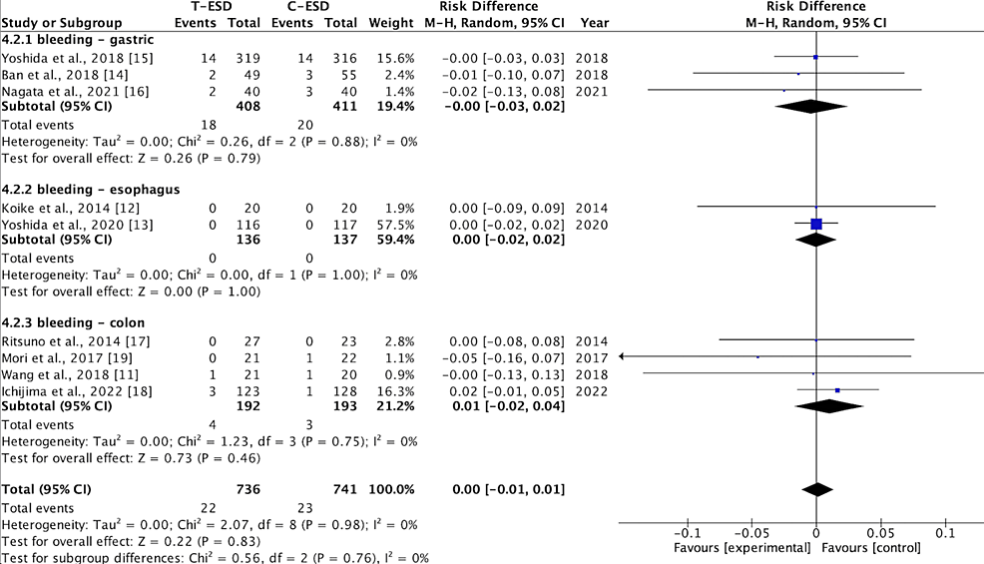
A forest plot of the bleeding rate: subgroup analysis T-ESD: traction-assisted endoscopic submucosal dissection with traction); C-ESD: conventional endoscopic submucosal dissection

In the subgroup analysis of the esophagus (RD: <0.00; 95% CI (-0.02, 0.02); p = 1.00; I2 = 0%), stomach (RD: <-0.00; 95% CI (-0.03, 0.02); p = 0.79; I2 = 0%), and colorectal region (RD: 0.01; 95% CI (-0.02, 0.04); p = 0.46; I2 = 0%), there was also no difference between C-ESD and T-ESD (Figure 9).

The GRADE analysis revealed a high certainty of evidence.

Discussion

This meta-analysis reveals that the T-ESD technique, when compared to C-ESD, has better efficacy when the aspect evaluated is the mean dissection time. This can be evidenced both in the grouped analysis and in the subgroup analysis for the esophageal and colorectal regions. Regarding safety, it is possible to identify a result equally favorable to T-ESD in the grouped and subgroup evaluations for the colorectal region when analyzing the perforation rate.

However, there was no difference in efficacy between the techniques when evaluating grouped or subgroup en bloc resection rates, as well as the mean dissection time for the stomach subgroup. In terms of safety, it was not possible to identify a difference in the grouped or subgroup analysis when the outcome assessed was the bleeding rate, as well as in the perforation rates for the esophagus and stomach subgroups.

Endoscopic submucosal dissection is a technique that allows lesions to be resected with protection of the lateral margins and in a monobloc fashion, even in larger lesions of the digestive tract. Although the C-ESD and T-ESD techniques have equivalent en bloc resection rates, the fact that we can perform traction on the lesion as an auxiliary method allows us to bring endoscopic-type movements closer to "laparoscopic" type movements [20,21]. In this way, tissue triangulation becomes possible, facilitating dissection with an endoluminal instrument that, until then, performed unidirectional movements. This can influence practicality, efficiency, and safety during the procedure [22]. Thus, this technique may make more endoscopists feel more encouraged to perform it.

The best results in mean dissection time identified for the esophagus and colorectal region can be explained by the fact that they are tubular organs, thus allowing better control of traction techniques when a device is placed on the contralateral wall. Likewise, as they vary little in caliber, the endoscopy device is parallel to the axis of these organs, facilitating dissection in both the frontal and rear views [23].

As far as safety is concerned, one of the biggest fears when carrying out the procedure is the occurrence of perforation, as this can lead to greater morbidity for patients and higher hospital costs. The traction method, by allowing better visualization of the submucosal and muscular layers, makes the procedure safer and reduces the risk of perforation, as occurred in the colorectal subgroup. 

The currently established traction techniques (clips, coils, and dental floss), regardless of which one is used, show the benefits of their use. Even so, it must be acknowledged that there has been variability in the traction method used and new methods have been described even more recently. Methods such as magnetic counter-traction and the suture pulley method have shown promising results in animal studies. The pulley and suture method was tested in a porcine model by 13 endoscopists, demonstrating a significant reduction in procedure time and the technical demands of ESD, especially among younger endoscopists [24]. Similarly, the magnetic counter-traction device reduced dissection time by almost half when performed on a porcine model with simulated lesions up to 30 mm in diameter [25].

The positive points of this meta-analysis are the inclusion of only RCTs, which constitute a 1A level of evidence; the low heterogeneity of most of the results analyzed was related to a rigorous methodology; and all the outcomes analyzed were evaluated by all the studies.

This study has some limitations. All the studies included were from Asia. On this continent, traditionally, a greater number of ESDs are performed, presenting a significant number of cases and a typical routine for endoscopists. This may interfere with defining the reproducibility of the method for other services. In addition, the studies included different sizes, morphologies, and locations of lesions in the different organs and different traction devices, which makes it more difficult to define specific situations where and which traction method may be more beneficial. 

## Conclusions

In conclusion, the meta-analysis underscores the positive impact of T-ESD over C-ESD, notably evident in the significant reduction of mean dissection time, particularly observed in the esophageal and colorectal regions. This efficiency boost is attributed to the traction techniques' ability to streamline endoscopic movements, thereby enhancing procedural practicality and efficacy. Moreover, the lower perforation rate observed in the colorectal subgroup highlights T-ESD's enhanced safety profile, facilitated by improved visualization of submucosal and muscular layers. Notably, both techniques demonstrate comparable en bloc resection rates and bleeding rates across all evaluated subgroups.

While acknowledging these positive outcomes, it's essential to recognize the challenges posed by the variability in traction techniques and lesion characteristics across studies, which can impact the determination of the optimal approach for different clinical scenarios. Nonetheless, the homogeneity of results and the inclusion of only RCTs reinforce the reliability of the findings. However, the concentration of studies in Asia and the diverse lesion characteristics underscore the need for caution in generalizing these results to other regions and settings. In essence, while T-ESD offers promising enhancements in procedural efficiency and safety, further research is necessary to ascertain its applicability across diverse clinical contexts and lesion types.

## Appendices

Appendix one 

**Table 4:** The quality of the evidence in the included studies reveals a high degree of certainty. T-ESD: traction-assisted endoscopic submucosal dissection; C-ESD: conventional endoscopic submucosal dissection; MD: mean deviation

Certainty assessment							Results summary							Importance
Number of studies	Study design	Risk of bias	Inconsistency	Indirectness	Imprecision	Other considerations	Number of patients		Effect			Certainty		
							T-ESD	C-ESD	Relative risk (RR) (95% CI)	Absolute (95% CI)				
Dissection time: subgroup analysis														
9	Randomized controlled trials	Not serious	Not serious	Not serious	Not serious	None	736	741	-	MD 10.65 lower (18.62 lower to 2.67 lower)	⨁⨁⨁⨁ High		Important	
Dissection Time: overall														
9	Randomized controlled trials	Not serious	Not serious	Not serious	Not serious	None	736	741	-	MD 10.75 lower (18.76 lower to 2.74 lower)	⨁⨁⨁⨁ High		Important	
Perforation rate: overall														
9	Randomized controlled trials	Not serious	Not serious	Not serious	Not serious	None	4/736 (0.5%)	21/741 (2.8%)	RR 0.30 (0.12 para 0.76)	20 fewer per 1.000 (from 25 fewer to 7 fewer)	⨁⨁⨁⨁ High		Important	
Perforation rate: subgroup analysis														
9	Randomized controlled trials	Not serious	Not serious	Not serious	Not serious	None	3/736 (0.4%)	21/731 (2.9%)	RR 0.23 (0.08 para 0.62)	22 fewer per 1.000 (from 26 fewer to 11 fewer)	⨁⨁⨁⨁ High		Important	
Bleeding rate: overall														
9	Randomized controlled trials	Not serious	Not serious	Not serious	Not serious	None	22/736 (3.0%)	23/741 (3.1%)	RR 0.96 (0.54 para 1.70)	1 fewer per 1.000 (from 14 fewer to 22 more)	⨁⨁⨁⨁ High		Important	
Bleeding rate: subgroup analysis														
9	Randomized controlled trials	Not serious	Not serious	Not serious	Not serious	None	22/736 (3.0%)	23/741 (3.1%)	RR 0.96 (0.54 para 1.70)	1 fewer per 1.000 (from 14 fewer to 22 more)	⨁⨁⨁⨁ High		Important	
En bloc resection: overall														
9	Randomized controlled trials	Not serious	Not serious	Not serious	Not serious	None	732/736 (99.5%)	731/741 (98.7%)	RR 1.00 (0.99 para 1.01)	0 fewer per 1.000 (from 10 fewer to 10 more)	⨁⨁⨁⨁ High		Important	
Enbloc resection: subgroup analysis														
9	Randomized controlled trials	Not serious	Not serious	Not serious	Not serious	None	734/736 (99.7%)	731/741 (98.7%)	RR 1.01 (0.99 para 1.03)	10 more per 1.000 (from 10 fewer to 30 more)	⨁⨁⨁⨁ High		Important	
